# Comparison of Performance, Meat Lipids and Oxidative Status of Pigs from Commercial Breed and Organic Crossbreed 

**DOI:** 10.3390/ani4020348

**Published:** 2014-06-19

**Authors:** Giuseppe Martino, Cecilia Mugnai, Dario Compagnone, Lisa Grotta, Michele Del Carlo, Francesca Sarti

**Affiliations:** 1 Faculty of Biosciences and Agro-Food Technologies and Environmental, University of Teramo, via C. Lerici 1, 64023, Mosciano S.A., Italy; E-Mails: gmartino@unite.it (G.M.); dcompagnone@unite.it (D.C.); lgrotta@unite.it (L.G.); mdelcarlo@unite.it (M.D.C.); 2 Department of Agricultural, Food and Environmental Science, University of Perugia, Borgo XX Giugno, 74, 06121, Perugia, Italy; E-Mail: francesca.sarti@unipg.it

**Keywords:** pig, rearing system, performance, meat quality, oxidative status

## Abstract

**Simple Summary:**

In recent years, the development of alternative rearing methods, capable of satisfying requests regarding product quality, yet also taking animal welfare and environmental protection into consideration, is becoming an increasingly important consumer demand. When pigs are reared in free range and organic systems, outdoor access is given to pigs; and such rearing conditions increases energy demand for activity and thermoregulation, which reduces growth rate, but improves some meat quality characteristics, such as leaner meat with lower C14:0 and higher C20:1n9, and oxidative stability.

**Abstract:**

The aim of this research was to determine the effect of rearing systems for pig production, as concerns performance, meat lipid content, the fatty acid profile, histidinic antioxidants, coenzyme Q10, and TBARs. One hundred pigs were assigned to one of three treatments: intensively reared commercial hybrid pig (I), free range commercial hybrid pig (FR) or organically reared crossbred pig (O), according to organic EU Regulations. I pigs showed the best productive performance, but FR and O increased: C20:1n9, Δ9-desaturase (C18) and thioesterase indices in meat. Lipid, dipeptides and CoQ10 appeared correlated to glycolytic and oxidative metabolic pathways. We can conclude that all studied parameters were influenced by the rearing system used, and that differences were particularly evident in the O system, which produced leaner meat with higher oxidative stability. In this respect, the organic pig rearing system promotes and enhances biodiversity, environmental sustainability and food quality.

## 1. Introduction

The increased interest in reducing intensity of pork production has led to the implementation of systems aware of animal welfare and without impairment of environmental sustainability, economy and food security [1]. Intensive rearing systems commonly use commercial hybrids selected for their rapid growth rate and adaptability to limited space allowance. However, these genotypes are characterized by low capacity to cope with certain environmental conditions due to their low immune-competence and high susceptibility to environmental stress. In contrast, alternative rearing systems require animals characterized by a slow growth rate and high adaptability to various environments, which may lead to favorable productive performance. In Italy, the extensive rearing system of pigs is quite diffuse and linked to autochthonous pig breeding traditions oriented towards typical meat products [2]. These already existing systems (autochthonous genotypes/outdoor rearing), can be easily adapted to organic pig production matching: consumers opinion, which needs to be adjusted to lower input farming approaches [3], Council Regulation 1804/99/EC [4] and the Network for Animal Health and Welfare in Organic Agriculture’s final recommendations [5]. The latter suggests the housing of local, slow-growing breeds for their higher rusticity and capacity to utilize outdoor areas and pasture.

The effect of the rearing system on meat quality is still under debate, and, in particular for pigs, conflicting evidences are found in the literature on the effect of rearing system on the meat quality [6,7,8,9,10,11,12,13]. The contradictory data are probably due to large variations in the design of the production systems [11]. In this context, however, few data were reported on oxidative stability with respect to histidinic antioxidant and CoQ_10_ content of meat. Moreover no information is available on differences between rearing systems for the activity of the Δ9-desaturase complex in the synthesis of the LCMUFA (Long Chain Monounsaturated Fatty Acids). Since the quality of the product is crucial for consumer health, the study reported in here was planned, in order to integrate the issue of a high quality product with those related to animal biodiversity and welfare, and environmental sustainability of livestock production. Thus, the aim of the present study was to evaluate performance, lipids, the fatty acid profile and different indices together with the oxidative status of pork meat produced by different rearing systems, using different analytical techniques.

## 2. Materials and Method

### 2.1. Animals, Rearing Systems and Productive Traits Procedures

Two hundred pigs of Commercial hybrid and 100 F1 pig of crossbreed Cinta Senese X Large White (CSXLW), were used for the study. Cinta Senese was selected for F1 pig production, because they are well adapted to outdoor environmental conditions as reported by Council Reg. 1804/99/EC [4].

All animals were reared at the facilities of Soc. Coop Agricoltura Nuova Cooperativa s.r.l. farm, located at Gessopalena (CH, 450 mt above sea level), in the Maiella mountains, Italy. All pigs (gender ratio 50:50), were held and fed in the same conditions from birth until 40 kg of bodyweight (indoor pen group housed; 5 pigs per pen/group). The experiments were performed in the 40–150 kg range of bodyweight. The experimental phase began in April and pigs were slaughtered in August (130 day of experimental phase).

Pigs were assigned to either of the following rearing systems:

- Intensive (I): 100 piglets of Commercial hybrid, housed in two (3.1 m^2^/pig, half concrete area, half metal slats) environmentally controlled buildings (22 °C and 60 to 70% relative humidity);
- Free Range (FR): 100 piglets of Commercial hybrid, were raised outdoors in a 1 ha extension land in two pens of 5000 m^2^ each (100 m^2^/pig of pasture), with access to igloos (4 × 2 × 1.4 m^2^/pigs) with barley straw bedding;
- Organic (O): 100 crossbred piglets (CSXLW) were raised outdoors in a 25,000 m^2^ extension land in two pens of 12,500 m^2^ each (250 m^2^/pig of pasture), according to Council Reg. 1804/99/EC [4], with access to igloos (4 × 2 × 1.4 m^2^/pigs) with barley straw bedding.

Two repetitions of 50 pigs each per group were carried out; for I pigs, the building was considered as repetition; whereas for outdoor reared pigs, the pen was considered as repetition.

Indoor pigs were placed in temperature-controlled buildings where temperatures did not fall below 18 °C. The average air temperature for the outdoor pigs during this trial was 21 °C (range: 3 to 38 °C) and the average relative humidity was 60%. The air temperature was within normal ranges during this study period.

From 23 to 70 kg BW, and from 70 to 150 kg BW, I, FR and O groups had free access to water and *ad libitum* access to the same organic grower and ﬁnisher diets (Table 1), certified by a National agency.

**Table 1:** Chemical composition (g/100 g DM) and Ingredients of diets.

Diets	Grower	Finisher
Chemical composition		
Crude protein	17.82	17.73
Ether extract	5.30	5.65
Crude fiber	5.33	3.92
Ash	5.21	5.49
Digestible energy *	3665.51	3772.88
Ingredients ^†^		
Barley, wheat, starch wheat flour, roasted soybean, corn, fats, molasses, sunflower extraction meal, calcium carbonate, dicalcium phosphate, sodium chloride.		

* (kcal/kg d.m.); ^†^ ingredients are presented in decreasing order.

Pigs were weighed and feed disappearance was recorded weekly. No castration of male piglets, teeth clipping, ear tattooing or tail docking was conducted.

### 2.2. Slaughter and Sampling Proceedings

When pigs achieved a BW of 150 ± 10.0 kg, they were slaughtered on two consecutive days (N = 150 pigs slaughtered/day, N = 50 per group replication) at the communal abattoir of Casoli (CH), situated less than 10 km from the breeding site. All travel organization followed the rules of Council Regulation (EC) No 1/2005 of 22 December 2004 [14]. FR and O pigs were brought inside the abattoir holding pens 16 h before slaughter, to ensure that no additional feed was consumed by the pigs. I pigs were slaughtered, 16 h after feed withdrawal.

All pigs were stunned electrically (300 V, 3 s) with a pair of stunning tongs, after which they were shackled by the hind leg and exsanguinated. Five minutes after exsanguination samples (N = 25 per replication/day per group) were taken with a cork bore from the *Longissimus dorsi* (LD) between 13th and 14th ribs after approximately 5 min of blooming time. Muscle cubes (0.5 cm^3^) were cut from the LD muscle sample. All fresh meat samples, were packed under modified atmosphere and stored at −80 °C till analysis. Muscle samples and measurements were taken from a total of 50 pigs/group.

### 2.3. Chemical Analysis of Meat

#### 2.3.1. Lipids, Fatty Acid Profile and Indices

Total meat lipids (samples of about 5 g) were extracted [15] in a homogenizer with 50 mL of 2:1 (*vol/vol*) chloroform-methanol, followed by filtration through Whatman No. 1 filter paper.

Lipid extract was trans methylated to fatty acyl methyl esters (FAMEs) at room temperature by using KOH 2 M in anhydrous methanol. Fatty acid methyl esters (FAMEs) composition was determined by gas chromatography using gas chromatograph (Fisions Mega 2 Carlo Erba, Gas Chromatograph, model HRGC, Milan, Italy) with flame ionization detection (FID) equipped with a VARIAN capillary column CP-SIL 88 of 100 m the carrier gas was hydrogen. Oven temperature programming was as follows: 160 °C held for 1 min; 175 °C at 4 °C/min, held for 28 min; 215 °C at 3 °C/min, held for 30 min; 160 °C at 10 °C/min.

The identification of fatty acids (FA) was based on the comparison of retention times of individual FA with standard mixture (Sigma-Aldrich) and FA values were expressed as percentage of the sum of total FA determined. The FA percentages were calculated with the Chrom-Card software.

The activities of Δ9-desaturase and elongase were estimated by relating the amount of product to that of precursor [16]. Particularly, the Δ9-desaturase (16) index was calculated as the relative amount (%) of palmitoleic acid (C16:1) with respect to C16:1 and palmitic acid (C16:0). The Δ9-desaturase (18) index was expressed as the relative amount of C18:1 (C18:1n7+C18:1n9) with respect to the total amount of C18:1 (C18:1n7+C18:1n9) and stearic acid (C18:0). Consequently, the Δ9-desaturase (16+18) index was calculated as the relative amount of the sum of C16:1, C18:1n7 and C18:1n9 *vs.* the amount of C16:1, C16:0, C18:1n7, C18:1n9, and C18:0. The elongase index was calculated as the ratio of C18:0 to C16:0. The thioesterase index was calculated as the ratio of C16:0 to myristic acid (C14:0).

#### 2.3.2. Antioxidant Compounds Analysis

Meat samples were analyzed for anserine and carnosine determination using the method from Aristoy and Toldrà [17], with minor modifications. A procedure for the determination of the histidinic antioxidants was optimized using ion-exchange chromatography on a Bonus RP 3.5 µm, 4.6 × 150 mm column. After removing blood, connective tissue, and subcutaneous fat, meat samples were weighed (2.0 ± 0.1 g) and homogenized with 20 mL of 0.01M trichloracetic acid (TCA) for about two minutes. The homogenate was then centrifuged at 4000 × g for 60 min at +4 °C. The supernatant was collected and filtered on 0.45 µm nylon filter. Before injection, samples underwent a 20 fold dilution in mobile phase. Optimal separation of carnosine and anserine was achieved using a Bonus RP 3.5 µm, 4.6 × 150 mm column in 10 mM acetate buffer at pH 5.0 plus 5% acetonitrile and using 5 mM of sodium Octanesulphonic acid as ion pairing agent with isocratic elution. The flow rate was 1 mL/min. Alanine-Phenylalanine was selected as internal standard because its chromatographic behavior is similar to carnosine and anserine but the retention time is in a “blank window” interval of the chromatogram obtained with meat samples extracts, where no peaks are observed. Optimal pH for the use of Ala-Phe was 5.0, at this pH peak tailing of the internal standard was absent and good separation of anserine and carnosine was obtained at retention times of, 5.8 min for Anserine and 6.4 min for carnosine. Due to its more hydrophobic nature Ala-Phe exhibited a retention time of 8.3 min. Calibration curves for anserine and carnosine obtained in the 0–100 µg/mL range with the use of internal standard were linear using a 20 µg/mL carnosine and anserine standard solution. These values were compared with those reported by Gil-Agustì *et al.* [18], with correlation coefficient r = 0.997.

Ubiquinone (CoQ_10_) analysis was carried out according to the method of Martino *et al* [19], with modification. Samples (2 g) were minced and homogenized in 10 mL of methanol containing 4% pyrogaroll; saponification at 80 °C for 15 min after adding 1 mL of 59% KOH solution, and extraction was done using 20 mL of hexane; after evaporation under a nitrogen stream, the extract was dissolved in 1 mL of methanol and 10 μL of solution was injected into the liquid chromatograph (Varian, Model 9010, Walnut Creck, CA, USA). For HPLC determination, an LC18 column (4.6 × 250 mm, 5 m) and UV detector (Varian, Model 9050, Walnut Creck, CA, USA), adjusted to 275 nm and detection set at 0.0001 AUFS, were used. For the separation, HPLC grade 2-propanol:methanol (40:60) mobile phase was employed at flow rate of 2 mL/min. For the determination of the CoQ_10_ an appropriate calibration curve was built with standard (Sigma, St Louis, MO, USA).

#### 2.3.3. Fat Oxidation

The extent of intra muscular fat oxidation was evaluated by measuring thiobarbituric acid reactive substances (TBARS) [20]. The TBA distillation method was performed as described by Tarladgis *et al.* [21] except that butylated hydroxytoluene (BHT) was added before homogenization. Meat samples (3.5 g) were homogenized with 50 mL of (5%) TCA and 500 µL of BHT, with Ultra Turrax T25 at 4000 × g, for 5 min. Two mL of 0.02 M TBA in acetic acid were added to 2 mL of distilled Homogenate. This solution was heated in a water bath at 80 °C for 1 h and then cooled for 10 min with cold tap water. The absorbance was determined by Perkin Elmer Lambda 20 UV/vis spectrophotometer a 534 nm against a blank containing 2 mL of distilled TCA and 2 mL of 0.02 M TBA solution. This value was calculated from standard curves and known dilutions.

### 2.4. Statistical Analysis

The effect of treatment were tested with the procedure SAS/GLM [22], using the following model:

yijk = μ + ai + ei

where μ = overall average; ai = fixed effect rearing system (i = 1 … 3); ei = error.

For each of the three rearing systems, two repetitions (50 pigs each rearing system/per replication for productive performance records; and 25 samples of *Longissimus dorsi* of pig for each rearing system per replication for chemical analysis), were used. In a preliminary analysis the effect of gender and replicate was verified; however, they didn’t reach the significance level and therefore these two factors and their interactions in the final analysis of the data, were not considered. The significance of differences was estimated by the multiple Student’s *t*-test. Differences were considered significant for *P* < 0.0001 and *P* < 0.05.

## 3. Results and Discussion

A total of 300 pigs (50 for each repetition/rearing system) completed the production period according to the experimental plan, and 150 (25 for each repetition/rearing system) *Longissimus dorsi* of pigs completed all the meat quality measurements.

### 3.1. Productive Performance

Independently of the rearing system, the overall performance of the pigs was good (Table 2). Mean daily gain was 847 g, and carcass dressing out was 81%. Although in I husbandry practice the mix of pigs in a confined space can result in significant stress reactions and aggressive behavior because of frustration or restriction of natural behavior [23,24], no mortality or health problems occurred during the experimental period, starting from 40 kg live weight until slaughter at a live weight around 150 kg.

**Table 2:** Effect of rearing system on productive performance of pigs.

Rearing System		I	FR	O	SED
Starting BW ^†^	Kg	39.42	39.48	37.18	5.15
ADG *	Kg	0.92	0.86	0.76	0.12
Feed:Gain Ratio		2.70 ^a^	3.11 ^b^	3.90 ^c^	0.90
Slaughter BW ^†^	Kg	159.02 ^b^	151.28 ^ab^	135.98 ^a^	4.02
Dressing out	%	83 ^b^	80 ^ab^	78 ^a^	2.39

N = 100/group; ^ac^ Means in the same row with the same superscript do not differ significantly (*P* < 0.05); ^†^ Body Weight; * Daily Weight Gain.

I pigs showed the best (*P* < 0.05) performance (Feed:Gain Ratio and slaughter BW). The main physical differences among treatments were in climate control and degree of exercise. Thus, the activity level of FR and O pigs is expected to rise respect I pigs. Close and Poorman [25] calculated an additional energy expenditure of 1.67 kcal ME/kg bodyweight per km walked in growing pigs. The higher energy requirements for activity and thermoregulation in outdoor pig rearing [26], therefore, increased requirements of FR and O pigs. Thus, as expected, O pigs had the higher (*P* < 0.05) Feed:Gain Ratio, even higher than the FR group [6,7,9], because of the larger access area and the lower potential for growth of Cinta Senese [27]. Accordingly, O pigs showed the lower (*P* < 0.05) slaughter BW (159.02 vs 151.28 and 78%, or O, FR and I, respectively) and dressing out percentage (78 *vs.* 80 and 83%, for O, FR and I, respectively).

### 3.2. Meat Characteristics

Lipid content, fatty acid profile and indices of *Longissimus dorsi* of pig are reported in Table 3.

**Table 3:** Effect of rearing system on lipid content (g/100 g of muscle), fatty acid profile (g/100 g of total lipids) and fatty acid indices of *Longissimus dorsi*.

Rearing System	I	FR	O	SED
Lipid content	4.23 ^c^	3.50 ^b^	2.16 ^a^	0.52
Fatty acid Profile				
C14:0	1.48 ^b^	1.56 ^b^	1.20 ^a^	0.24
C16:0	27.01	26.66	26.77	1.51
C18:0	13.03	12.57	12.77	1.36
SFA	41.52	40.79	40.74	2.33
C16:1n7	3.16	3.47	3.42	0.57
C18:1n7	3.87	4.07	4.08	0.42
C18:1n9	42.32	41.50	41.02	2.58
C20:1n9	0.86 ^a^	1.24 ^b^	1.62 ^c^	0.32
MUFA	49.35	49.03	48.52	2.76
C18:2n6	6.26	7.28	7.05	1.59
C18:3n3	0.74	0.70	0.67	0.12
PUFA	7.00	7.97	7.73	1.57
Fatty acid indices				
Δ9-desaturase (C16) ^1^	10.47	11.52	11.33	0.98
Δ9-desaturase (C18) ^2^	458.46 ^a^	478.38 ^b^	478.88 ^b^	2.50
Δ9-desaturase (C16+C18) ^3^	55.21	55.56	55.10	0.19
Elongase ^4^	0.48	0.47	0.48	0.12
Thioesterase ^5^	18.25 ^a^	17.09 ^a^	22.31 ^b^	2.23

N = 50/group; ^ac^ Means in the same row with the same superscript do not differ significantly (*P* < 0.05); ^1^ Calculated as 100 × [16:1n-9/(16:1n-9 + 16:0)]; ^2^ Calculated as 100 × [(18:1n7 + 18:1n-9)/(18:1n7 + 18:1n-9 + 18:0)]; ^3^ Calculated as 100 × [(16:1n-9 + 18:1n7 + 18:1n-9)/(16:1n-9 + 16:0 + 18:1n7 + 18:1n-9 + 18:0)]; ^4^ Calculated as 16:0/14:0; ^5^ Calculated as 18:0/16:0.

A significant (*P* < 0.001) effect of rearing system was found for muscle lipid content with I pigs having the higher amount followed by FR and O pigs. This was due to the greater energy demand (activity and temperature regulation) of outdoor reared pigs, thus producing leaner meat.

Regarding the effect of rearing system on meat lipid content, literature data are conflicting. Our results agree with Enfalt *et al.* [6] and Sather *et al.* [7] that found increased carcass lean meat contents in free-range pigs. Also Bee *et al.* [28] found a decreased lipid content in outdoor/free-range reared pigs.

On the contrary, other Authors [12,29,30] found a greater lipid content of pigs reared with outdoor access compared with the confined.

Considering fatty acid profile, rearing system showed significant (*P* < 0.05) effect only on myristic (C14:0) and gondoic acids (C20:1n9) content. C14:0 was found higher (*P* < 0.05) in I and FR; whereas for C20:1n9: O > FR > I, (*P* < 0.05). With respect to C14:0, Bellizzi and Coworkers [31], found a positive correlation with coronary heart disease mortality. Moreover, the same Authors noted that myristic acid was the major cause of hypercholesterolemia induction. This effect was related both to repression of hepatic low density lipoprotein (LDL) receptor synthesis and to direct stimulation hepatic LDL synthesis. C20:1n9 is a long-chain monounsaturated fatty acids (MUFA), biosynthesized from oleic acid, and was found to alleviate metabolic syndrome partly by regulating genes involved in lipid metabolism, energy expenditure, and inflammation in obese mice [32] and to lower risk of HIV transmission through breast milk [33]. Moreover some Authors [34,35] suggested a correlation between plasma levels of long-chain MUFA and metabolic parameters as hyperglycemia and hyperlipidemia.

The rearing system was a significant source of variation (*P* < 0.05) for Δ9-desaturase (C18), while elongase index appeared to be affected by genotype. These data are in accordance with Zhang *et al.* [36], which found a higher thioesterase index in Landrace, Yorkshire, Hampshire, and Spotted pigs with respect to Duroc and Chester White pigs. Thioesterase in the fatty acid synthase complex terminates the fatty acid synthesis and the release of the newly synthesized fatty acids. Both C14-acyl ACP (Acil Carrier Protein) and C16- acyl ACP are substrates for thioesterase, C16:0 being the major product. The ratio of C16:0 to C14:0 was utilized to reflect the selective cleavage of thioesterase on C14-acyl ACP or C16-acyl ACP; the greater the thioesterase index, the less cleavage of C14-acyl ACP.

In Table 4 the effect of rearing system on anserine, carnosine, CoQ10 and TBARs of meat contents is presented.

The anserine values showed a decreasing trend in meats: I > FR > O pigs, (*P* < 0.05). This trend is in agreement with data published in the literature, which suggests that the anserine content of meat is closely related to the glycolytic metabolism of muscle [37]. Accordingly, outdoor reared pigs showed the lower content, probably due to the more pronounced oxidative metabolism for higher activity.

**Table 4:** Effect of rearing system on histidyl dipeptides, TBARs and CoQ10 contents of *Longissimus dorsi* of pork.

Rearing system		I	FR	O	SED
Anserine	ppm	825.34 ^b^	567.56 ^ab^	275.41 ^a^	551.60
Carnosine	ppm	8594.50 ^b^	8746.63 ^b^	3581.25 ^a^	1006.82
CoQ10	ppm	7.19 ^a^	9.51 ^b^	17.99 ^b^	1.21
TBARs	ppm	0.32 ^c^	0.23 ^b^	0.16 ^a^	0.06

N = 50/group; ^ac^ Means in the same row with the same superscript do not differ significantly (*P* < 0.05).

Considering carnosine content, I and FR pigs exhibited higher values (*P* < 0.0001). Carnosine reacts with simple sugars and blocks the glycation process of proteins, and therefore behaves like a competitive acceptor in the glycoxidation reaction. Some studies have shown that carnosine and its related molecules are capable of acting as antioxidants [38]. Our results are consistent with previous studies [39] demonstrating that white muscle had higher carnosine content than dark muscle. Sewell *et al.* [40] found a strong positive correlation between carnosine content and type II fiber surface area in the equine middle gluteal muscle. These authors showed that carnosine can contribute as much as 46% to the buffering of H+ produced by equine type IIB fibers and concluded that this dipeptide is important in stabilizing the pH in anaerobic contraction.

Our data on O carnosine content suggest a kind of genotype effect. In fact, carnosine content is affected not only by kinetic activity, fiber type, but also by breed, gender, age and breeding [41]. Kanok-Orn *et al* [42], demonstrated that carnosine contents in chicken fresh meat of both breast and thigh meats were signiﬁcantly different among breeds. Oxidative intermediate and oxidative slow twitch muscle fibers have been found in the *Longissimus dorsi* muscle of wild pigs compared to domesticated pig breeds [43]. Thus, the lower concentration of anserine in O pigs is probably correlated to more pronounced oxidative metabolism, both for the performed kinetic activity and for the crossbreed used. This may explain the similar content of carnosine found in I and FR meat with respect to O meat.

Significantly higher values for CoQ_10_ were found in O and FR pigs than in I pigs (*P* < 0.05). It is now well established that CoQ_10_ (Coenzyme Q_10_) is a lipid soluble, endogenous hydroxybenzoquinone compound found in the majority of aerobic organisms [44] in two redox forms, and varying length of the isoprenoid side chain. Coenzyme Q_10_ plays an important role as an essential electron carrier in the mitochondrial respiratory chain linked to cellular oxidative metabolism and that is able to prevent lipid peroxidation in most subcellular membranes. Accordingly, we found greater value of CoQ_10_ in O and FR pigs, with outdoor access and increased kinetic activity.

According to CoQ_10_ and lipid contents (that represent the substrate for peroxidative processes), we found TBARS content (*P* < 0.05) in meat, in decreasing order: I, FR and O.

The outdoor rearing system induced the decrease of anserine dipeptide meat content, accompanied by the increase in CoQ_10_ meat content. In fact, when oxidative stress is reported, CoQ10 biosynthesis is increased [45].

TBARS content followed the same trend (*P* < 0.05) found for lipids; higher values were found for I, followed by FR and O meat samples. Lipid oxidation is one of the primary causes of quality deterioration in meat and generates compounds potentially dangerous for human health. The conversion of muscle to edible meat after slaughter can unbalance the equilibrium between pro-oxidative and anti-oxidative factors, resulting in initiation and propagation of lipid oxidation [46].

Possibly, the larger amount of CoQ10 together with the lower oxidation substrate (lipid), repressed the formation of end products of oxidation in O meat, thus reducing food deterioration [47]. Anyhow, TBARS content of meat for all studied systems was very low.

## 4. Conclusions

Extensive rearing systems of farm animals are perceived by consumers as strongly linked to health, animal welfare, sustainability, and safety; for these reasons, free range and organic rearing systems are highly desirable. Organic farming is the most regulated of the alternative rearing systems, with minimum environmental impact, increased welfare of production animals, and with no use of polluting agents, including antibiotics [48]. The issue is whether these new systems indeed improve welfare and sustainability of farmed pigs and whether, since they replace systems that already produce high-quality meat, they affect production characteristics and meat. On the basis of the reported results, we can conclude that rearing system influences all studied parameters, and when outdoor access is given to pigs, activity and thermoregulation reduce growth rate, but improve some meat quality characteristics (lean meat with lower C14:0, and higher C20:1n9 and oxidative stability). It is evident that for lipid, thioesterase and antioxidant dipeptides of meat, differences could be attributed also to the used genotype.

Differences were particularly evident in O system, with greater outdoor availability and the used crossbred. In our case the CSxLW crossbreed in organic rearing system produced very lean meat with higher oxidative stability. Thus, the aims of the organic production system were fully achieved, both the rearing and diffusion of local genotypes, and the improvement of the role of agriculture in environment preservation.

Further studies should be carried out in the future to deeply investigate the relationships between rearing system, used genotype, muscle energy metabolism and meat quality.
